# Autoradiography validation of novel tau PET tracer [F-18]-MK-6240 on human postmortem brain tissue

**DOI:** 10.1186/s40478-019-0686-6

**Published:** 2019-03-11

**Authors:** Cinthya Aguero, Maeva Dhaynaut, Marc D. Normandin, Ana C. Amaral, Nicolas J. Guehl, Ramesh Neelamegam, Marta Marquie, Keith A. Johnson, Georges El Fakhri, Matthew P. Frosch, Teresa Gomez-Isla

**Affiliations:** 1 Department of Neurology, Massachusetts General Hospital, WACC, Suite 715, 15th Parkman St., Boston, MA 02114 USA; 2 MassGeneral Institute for Neurodegenerative Disease, Charlestown, MA USA; 3 Center for Advanced Medical Imaging Sciences, Division of Nuclear Medicine and Molecular Imaging, Department of Radiology, Massachusetts General Hospital, Harvard Medical School, Boston, MA USA; 4 AP-HP, Department of Nuclear Medicine, Pitié-Salpêtrière Hospital, Sorbonne University, UPMC Paris 06, CNRS UMR 7371, INSERM U1146, 75013 Paris, France; 5 Department of Radiology, Massachusetts General Hospital, Boston, MA USA; 6 C.S. Kubik Laboratory for Neuropathology, Massachusetts General Hospital, Boston, MA USA

## Abstract

[F-18]-MK-6240, a novel tau positron emission tomography (PET) tracer recently discovered for the in vivo detection of neurofibrillary tangles, has the potential to improve diagnostic accuracy in the detection of Alzheimer disease. We have examined regional and substrate-specific binding patterns as well as possible off-target binding of this tracer on human brain tissue to advance towards its validation. We applied [F-18]-MK-6240 phosphor screen and high resolution autoradiography to postmortem samples from patients with a definite pathological diagnosis of Alzheimer disease, frontotemporal lobar degeneration-tau (Pick’s disease, progressive supranuclear palsy and corticobasal degeneration), chronic traumatic encephalopathy, frontotemporal lobar degeneration-Tar DNA-binding protein 43 (TDP-43), dementia with Lewy bodies, cerebral amyloid angiopathy and elderly controls free of pathologic changes of neurodegenerative disease. We also directly compared the binding properties of [F-18]-MK-6240 and [F-18]-AV-1451 in human tissue, and examined potential nonspecific binding of both tau tracers to monoamine oxidases (MAO) by using autoradiography in the presence of selective monoamine oxidase A (MAO-A) and monoamine oxidase B (MAO-B) inhibitors. Our data indicate that MK-6240 strongly binds to neurofibrillary tangles in Alzheimer disease but does not seem to bind to a significant extent to tau aggregates in non-Alzheimer tauopathies, suggesting that it may have a limited utility for the in vivo detection of these pathologies. There is no evidence of binding to lesions containing β-amyloid, α-synuclein or TDP-43. In addition, we identified MK-6240 strong off-target binding to neuromelanin and melanin-containing cells, and some weaker binding to areas of hemorrhage. These binding patterns are nearly identical to those previously reported by our group and others for [F-18]-AV-1451. Of note, [F-18]-MK-6240 and [F-18]-AV-1451 autoradiographic binding signals were only weakly displaced by competing concentrations of selective MAO-B inhibitor deprenyl but not by MAO-A inhibitor clorgyline, suggesting that MAO enzymes do not appear to be a significant binding target of any of these two tracers. Together these novel findings provide relevant insights for the correct interpretation of in vivo [F-18]-MK-6240 PET imaging.

## Introduction

The recent development of several novel positron emission tomography (PET) tracers tailored to detect tau in the brain has opened the opportunity of using them to improve diagnostic accuracy in Alzheimer disease (AD) and related tauopathies, and to allow reliable quantification of brain tau burden and tracking of disease progression by in vivo neuroimaging [15, 35].

Emerging data from early studies -including our own- on postmortem material with the most validated thus far, [F-18]-AV-1451 (T807, Flortaucipir), have shown that this ligand binds with strong affinity to paired helical filament (PHF)-tau aggregates in AD brains and those that form as a function of age [20–22, 27, 35], closely matching the stereotypical spatiotemporal progression of neurofibrillary tangles (NFT) as described by Braak [3]. In agreement with these observations, patients clinically diagnosed with dementia of AD type and mild cognitive impairment (MCI) exhibit significantly higher in vivo [F-18]-AV-1451 retention than cognitively normal individuals in regions that are known to contain an elevated burden of tau lesions in AD [4, 6, 7, 11, 16, 25, 28, 33]. The overall utility of this tracer for in vivo selective and reliable detection of tau aggregates in non-AD tauopathies, however, seems very limited with the exception of certain tau mutations causing frontotemporal lobar degeneration (FTLD) characterized by tau aggregates [26] that contain all six isoforms of tau (three-repeat (3R) and four-repeat (4R)) [14] with PHF ultrastructure resembling NFT found in AD. We and others have shown that [F-18]-AV-1451 has low affinity for tau aggregates that contain primarily 4R tau with straight filament ultrastructure that predominate in tauopathies such as progressive supranuclear palsy (PSP), corticobasal degeneration (CBD), and most cases of FTLD. We also demonstrated the existence of robust [F-18]-AV-1451 off-target binding to melanin- and neuromelanin-containing cells and some weaker binding to blood components [21, 22]. Controversy exists as to whether AV-1451 may also exhibit significant nonspecific binding to MAO enzymes [12, 15, 17, 30], as it has been recently demonstrated for other tau PET tracers like THK-5351 [13, 24].

Several second-generation tau tracers have more recently been reported. The one that has garnered most attention and is largely considered to have most promise is [F-18]-MK-6240 from the Merck Translational Biomarkers team [15, 32]. To date, very limited information is available about the binding properties of this tracer. Merck’s researchers performed in vitro binding screens against a wide panel of known receptor, transporter, and enzyme targets but conducted only limited autoradiography studies on AD and control brain specimens [15]. Yet, this ligand is quickly making its way into observational studies and clinical trials. Thus, a comprehensive postmortem validation of MK-6240 is critical for determining its usefulness for antemortem diagnosis and staging of AD and other tauopathies, and to understand exactly what [F-18]-MK-6240 PET positivity means in terms of neuropathological substrate. Because MK-6240 was initially screened in vitro for NFT binding affinity using AD brain homogenates rich in NFT and an amyloid plaque tracer for counterscreening [15], and seemed to bind to the same site as AV-1451 in that tissue material, we predicted that, just like AV-1451, MK-6240 would exhibit high binding affinity and selectivity for PHF-tau lesions relative to non-PHF-tau lesions, Aβ deposits and α-synuclein and transactive response DNA binding protein-43 (TDP-43) aggregates.

To validate the site/s of MK-6240 binding and determine whether there is off-target binding, we examined the regional and substrate-selective autoradiograhic patterns of [F-18]-MK-6240 in postmortem brain, retina and skin tissue samples. Cases with a definite pathological diagnosis of AD, FTLD-tau (PiD, PSP, CBD), chronic traumatic encephalopathy (CTE), FTLD-TDP-43, dementia with Lewy bodies (DLB), cerebral amyloid angiopathy (CAA), metastatic melanoma, brain hemorrhages, and elderly controls free of neurodegenerative diseases were studied. Most of these cases had also been included in our previous validation studies of [F-18]-AV-1451 [21, 22], giving us the opportunity to directly compare the autoradiographic binding patterns of both tracers in comparable tissue samples.

## Materials and methods

### Tissue samples

Postmortem brain, retina and skin tissue samples from the Massachusetts and the Boston University Alzheimer’s Disease Research Centers Neuropathology cores were included in this study. Autopsies were performed according to standardized protocol [31] and tissue collection and use was approved by the local Institutional Review Boards. A summary of the demographic characteristics of the cases studied is shown in Table 1.

**Table 1:** Participants’ characteristics

ID#	Clinical diagnosis	Pathological diagnosis	Age at death (yrs)	Gender	PMI (hrs)	Braak & Braak (NFT)	CERAD score (neuritic plaques)	NIA-Reagan Institute criteria
#1	CTL	Normal adult brain	86	M	10	I	none	LP
#2	CTL	Normal adult brain	73	F	20	I	none	LP
#3	CTL	Normal adult brain	101	F	22	II	A	LP
#4	AD	AD	96	F	20	V	C	HP
#5	AD	AD	78	F	18	VI	C	HP
#6	AD	AD	97	F	12	VI	A	HP
#7	AD	AD	87	F	12	IV	B	IP
#8	AD	AD	60	M	24	VI	C	HP
#9	AD	AD	82	F	6	V	B	HP
#10	AD	AD	69	F	4	VI	C	HP
#11	AD	AD	70	M	6	V	C	HP
#12	AD	AD	66	F	2	VI	C	HP
#13	AD	AD	66	F	10	VI	C	HP
#14	AD	AD	97	F	24	V	C	HP
#15	AD	AD	81	M	7	IV	C	IP
#16	FTLD	AD/CAA	66	M	16	V	B	HP
#17	Memory, speech and gait difficulties, hand tremor	Diffuse CAA (D23N Iowa APP mutation)	45	M	N/A	I	none	LP
#18	AD	AD/Metastatic melanoma	75	M	35	V	B	IP
#19	FTLD	PiD, type A	61	M	19	N/A	N/A	N/A
#20	FTLD	PiD	62	M	19	I	A	LP
#21	FTLD (P301L Mutation)	FTLD	71	F	4	I	none	LP
#22	FTLD	DLDH	65	M	24	I	none	LP
#23	CBD	CBD	80	M	6	I	none	LP
#24	PSP	PSP	69	M	45	II	none	LP
#25	PSP	PSP	68	M	48	II	none	LP
#26	PSP	PSP	78	M	11	II	none	LP
#27	PSP	PSP	73	M	12	II	none	LP
#28	PSP	PSP	63	F	12	II	none	LP
#29	CTE	CTE (CTE stage II-III) [23]	25	M	27	0	none	LP
#30	CTE	CTE (CTE stage III)	56	M	N/A	II	none	LP
#31	CTE	CTE (CTE stage III)	46	M	72	III	none	LP
#32	CTE	CTE (CTE stage IV)	65	M	N/A	II	A	LP
#33	CTE	CTE (CTE stage III)	58	M	N/A	III	none	LP
#34	DLB	LBD	62	M	24	N/A	none	N/A
#35	DLB	LBD, brainstem predominant	76	M	17	II	none	LP
#36	PDD	LBD (Braak stage 4/6)	83	M	9	III	none	LP
#37	MSA	MSA, cerebellar type (MSA-C)	60	F	32	II	none	LP
#38	FTLD	FTLD-TDP-43, type A	69	F	16	I	none	LP
#39	FTLD	FTLD-TDP-43	55	M	14	I	none	LP
#40	FTLD	FTLD-TDP-43	68	M	49	I	none	LP
#41	FTLD	FTLD-TDP-43	64	M	12	I	none	LP
#42	Headache	Subarachnoid hemorrhage	92	F	N/A	IV	A	IP
#43	N/A	Parenchymal hemorrhage	75	M	N/A	I	none	LP

*Abbreviations: AD* Alzheimer’s disease, *APP* amyloid precursor protein, *CAA* Cerebral amyloid angiopathy, *CBD* Corticobasal degeneration, *CERAD* Consortium to establish a registry for Alzheimer’s disease, *CTL* Control subject, *DLB* Dementia with lewy bodies, *DLDH* Dementia lacking distinctive histopathology, *F* Female, *FTLD* Frontotemporal lobar degeneration, *HP* High probability, *IP* Intermediate probability, *LP* Low probability, *M* Male, *MSA* Multiple systemic atrophy, *NFT* Neurofibrillary tangles, *NIA* National Institute of Aging, *N/A* Non available, *PiD* Pick’s disease, *PMI* Postmortem interval, *PSP* Progressive supranuclear palsy, *TDP-43* TAR DNA binding protein 43

Histological evaluation of each case was routinely performed on a specific set of 19 blocked regions representative for a spectrum of neurodegenerative diseases. All paraffin-embedded blocks were stained with Luxol fast blue and hematoxylin and eosin (LH&E), while selected blocks were routinely stained for Bielschowsky silver stain and Aβ, α-synuclein, ubiquitin, TDP-43 and phospho-tau immunoreactivity. Blocks of frozen brain tissue containing hippocampal formation, entorhinal cortex (EC), frontal, parietal, temporal and occipital cortices, cerebellum, basal ganglia and midbrain were cut into sections 10 μm-thick in a cryostat (Thermo-Shandon SME Cryostat), mounted on Histobond adhesion slides (StatLab, TX) and used for [F-18]-MK-6240 phosphor screen and nuclear emulsion high resolution autoradiography, followed by immunohistochemistry using appropriate antibodies in each case.

### [F-18]-MK-6240 phosphor screen autoradiography

[F-18]-MK-6240 was synthesized as previously described [8]. Autoradiography experiments were performed using [F-18]-MK-6240 aliquots from material prepared from in vivo imaging on the same day, and following our previously published protocol [22]. In brief, 10 μm-thick frozen brain sections were fixed in 100% methanol at room temperature for 20 min and then transferred to a bath containing high specific activity [F-18]-MK-6240 in 10 mM PBS with a radioactivity concentration of approximately 10 μCi/ml. Adjacent brain slices were placed in a bath that was identical in all aspects except that unlabeled MK-6240 was added to yield 500 nM chemical concentration, a blocking condition sufficient to saturate essentially all available specific binding sites of tau [35]. Additional adjacent slices were also incubated in separate baths containing either [F-18]-MK-6240 or [F-18]-AV-1451 with a radioactivity concentration of approximately 10 μCi/ml and 20 μCi/ml respectively, and selective MAO-A (clorgyline) and MAO-B (deprenyl) inhibitors (Sigma-Aldrich) were added at a competing concentration of 1 μM to evaluate potential displacement of [F-18]-MK-6240 and [F-18]-AV-1451 binding signals. After incubation for 60 min, racks of slides were removed from the respective radioactive solutions and briefly incubated in a series of wash baths to remove unbound radiotracer. Wash solutions and incubation times were: 10 mM PBS for 1 min, 70% ethanol/30% PBS for 2 min, 30% ethanol/70% PBS for 1 min, and lastly 100% 10 mM PBS for 1 min. Racks were removed from the final wash solution and slices were allowed to air dry before transfer of the slides to a storage phosphor screen (MultiSensitive Phosphor Screen, PerkinElmer Life and Analytic Sciences, Shelton, CT) that had been photobleached immediately prior by exposure on a white light box for a minimum of 15 min. The slides and phosphor screen were enclosed in an aluminum film cassette and set in a dark area away from sources of radioactivity for the duration of the overnight exposure period. Under dim lighting conditions, the cassette was opened and the slides removed from the exposed screen, which was mounted to the carousel of the digital imaging system (Cyclone Plus Storage Phophor Scanner, PerkinElmer Life and Analytic Sciences). Scanning of screens was controlled by the manufacturer’s OptiQuant software package using the highest available resolution of 600 dpi (approximately 42 μm sampling interval). Digital images were saved in uncompressed form at full resolution and pixel depth. Images from adjacent brain slices incubated in the unblocked (high specific activity [F-18]-MK-6240 only) and blocking ([F-18]-MK-6240 plus 500 nM unlabeled MK-6240) conditions were compared to determine total and non-specific binding of [F-18]-MK-6240 in the tissue.

### [F-18]-MK-6240 high resolution nuclear emulsion autoradiography and immunohistochemistry

To obtain autoradiographic information at the cellular resolution level, frozen cryostat sections, adjacent to those used for phosphor screen autoradiography, were coated with a liquid photographic emulsion following our previously published protocol [5, 9, 22, 34]. Immunohistochemistry was then performed on the nuclear emulsion-dipped sections. First the sections were washed for 5 min with PBS, then incubated with 2.5% normal horse blocking serum for 20 min, followed by the appropriate primary antibody - anti-tau PHF-1 (1:100, mouse, kind gift of Dr. Peter Davies), anti-Aβ (1:500, mouse, clone 6F/3D, Dako), anti α-synuclein (1:100, mouse, Zymed) or anti-phospho TDP-43 (pS409/410) (1:3000, mouse, Cosmo Bio CO) - for 40 min at 37 °C, washed with PBS twice for 2 min, and then incubated with the secondary antibody (ImmPRESS™ anti-mouse IgG (Vector Laboratories product MP-2400, Burlingame, CA) or ImmPRESS™ anti-rabbit Ig (Vector Laboratories product MP-7401, Burlingame, CA)) for 40 min at 37 °C. The sections were washed again with PBS twice for 2 min, and developed with DAB solution (Vector Laboratories product SK-4100). H&E was used for counterstaining. Photomicrographs were obtained on an upright Olympus BX51 (Olympus, Denmark) microscope using visible light.

## Results

### [F-18]-MK-6240 phosphor screen autoradiography

**Fig. 1:**
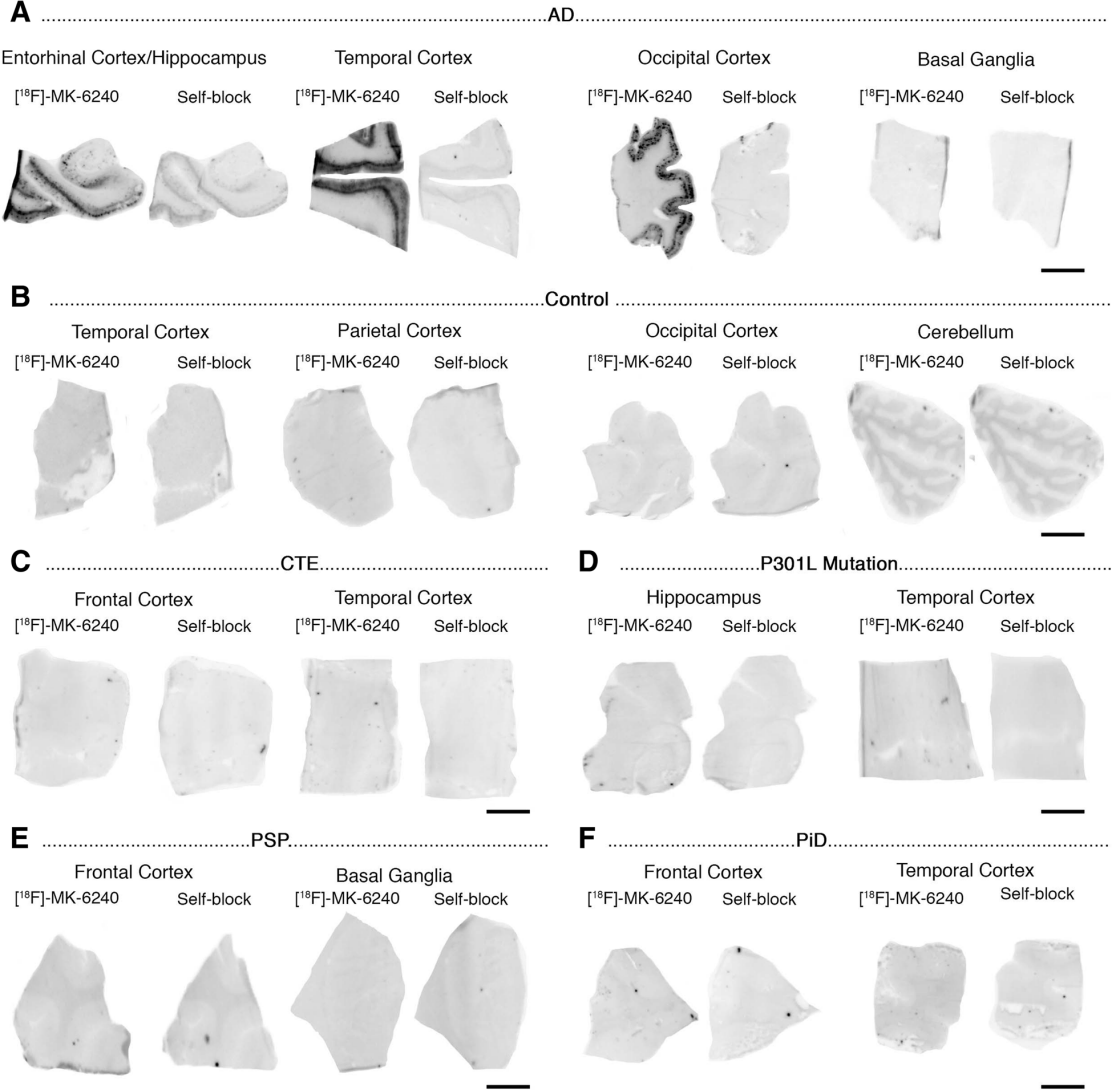
[F-18]-MK-6240 phosphor screen images of brain slices from AD (#5, #7, #9, #16) (**a**), control (#1, #2) (**b**), CTE (#32, #33) (**c**), P301L mutation carrier (#21) (**d**), PSP (#25) (**e**), and PiD (#20) (**f**) cases. A strong [F-18]-MK-6240 binding was observed in cortical regions containing tangles from AD brains. No signal was detected in basal ganglia, a region free of tangles. The signal was blocked by adding unlabeled MK-6240. Slices from a control case free of pathology did not show detectable [F-18]-MK-6240 binding (**b**). [F-18]-MK-6240 binding was not detectable either in non-PHF tau-containing slices from CTE (**c**), P301L mutation carrier (**d**), PSP (**e**) and PiD (**f**) cases. Abbreviations: AD = Alzheimer’s disease; CTE = chronic traumatic encephalopathy; PSP = progressive supranuclear palsy; PiD = Pick’s disease. Scale bar = 1 cm

**Fig. 2:**
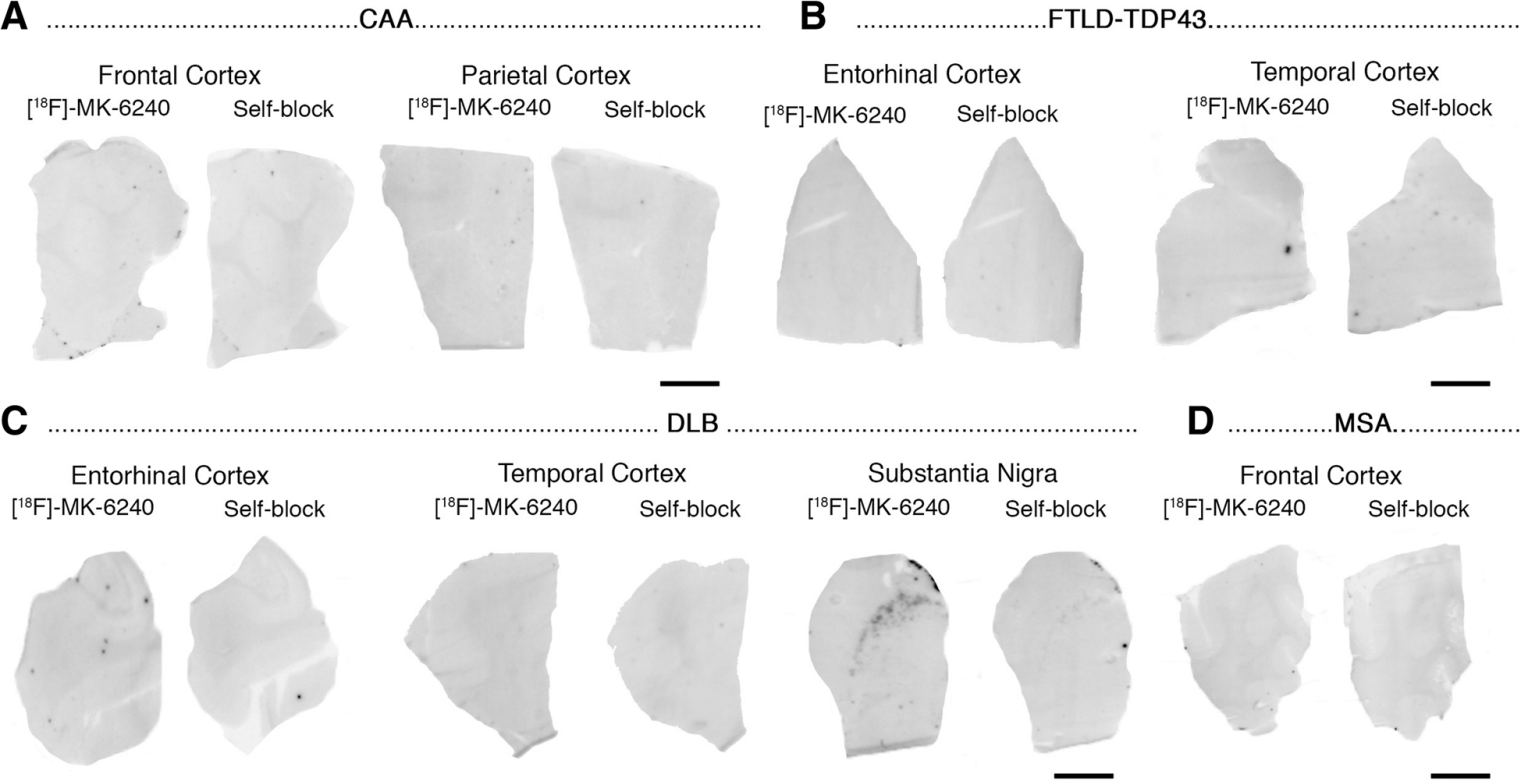
[F-18]-MK-6240 phosphor screen images of brain slices from a CAA carrier of the D23N Iowa APP mutation (#17) (**a**), FTLD TDP-43 (#40, #41) (**b**), LBD (#36) (**c**), and MSA (#37) (**d**) cases. No [F-18]-MK-6240 binding was detected in slices containing CAA lesions (**a**), TDP-43 inclusions (**b**), Lewy bodies (**c**) and glial α-synuclein inclusions (**d**). Strong [F-18]-MK-6240 signal was observed in the region corresponding to the substantia nigra (off-target) in all cases studied regardless of their pathological diagnosis (**c**). Abbreviations: APP = amyloid precursor protein; CAA = cerebral amyloid angiopathy; TDP-43 = TAR DNA binding protein 43; DLB = dementia with Lewy bodies; MSA = multiple system atrophy. Scale bar = 1 cm

**Fig. 3:**
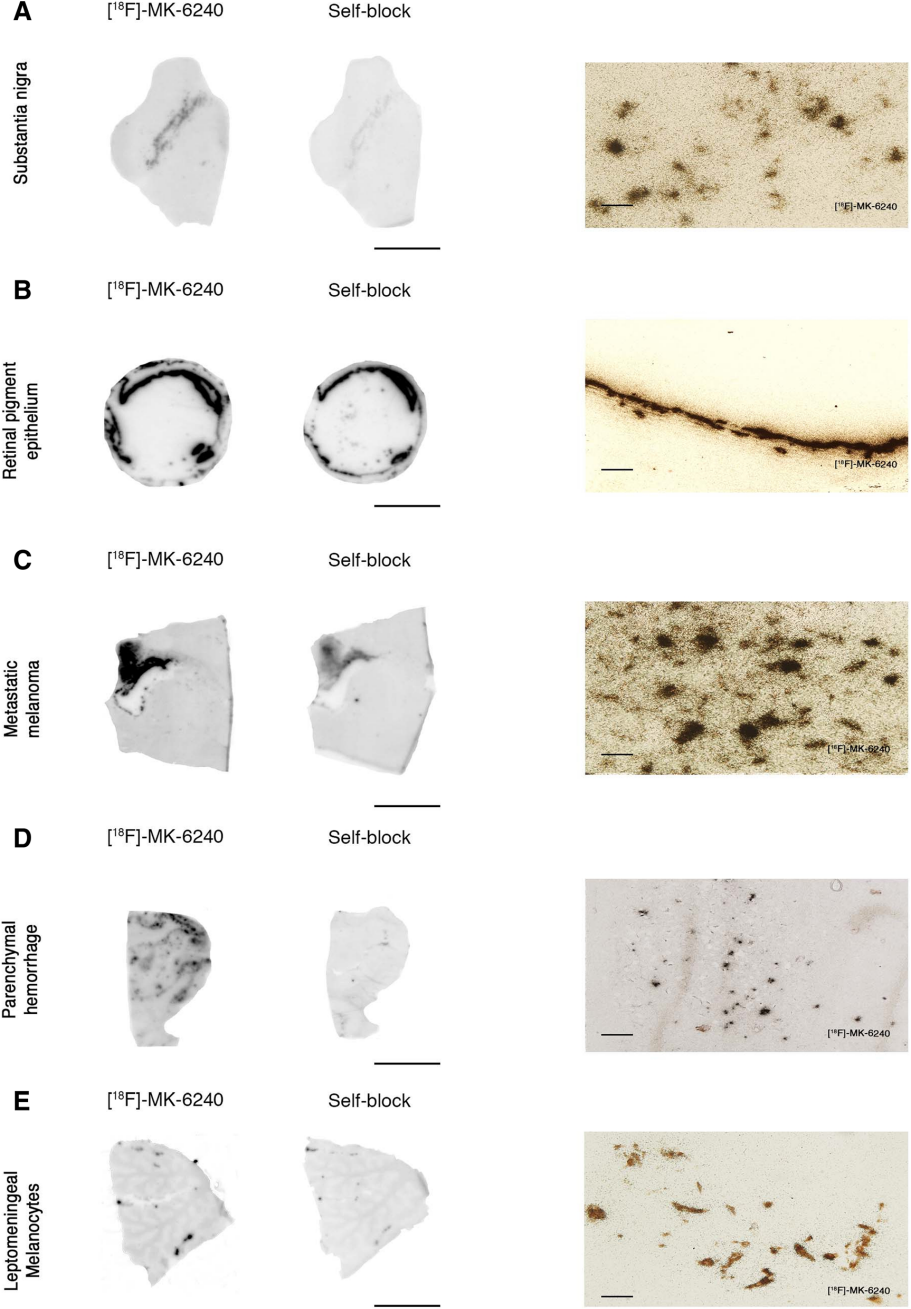
[F-18]-MK-6240 phosphor screen and high-resolution autoradiography images of slices containing substantia nigra in a control case (#2) (**a**), retinal pigment epithelium (**b**) in an AD case (#11), metastatic melanoma (#18) (**c**), parenchymal hemorrhagic lesions (#43) (**d**) and extracutaneous meningeal melanocytes in the cerebellum of an AD case (#8) (**e**). [F-18]-MK-6240 phosphor screen autoradiography images are displayed in (**a**-**e**), left and middle panels. [F-18]-MK-6240 high resolution autoradiography images are displayed in (**a**-**e**), right panel. Strong [F-18]-MK-6240 binding was observed in neuromelanin-containing neurons of the substantia nigra (**a**), melanin containing granules in the retinal pigment epithelium (**b**), malignant melanocytes from a metastatic melanoma (**c**), and extracutaneous meningeal melanocytes (**e**). [F-18]-MK-6240 binding was noticed in association with intraparenchymal hemorrhagic lesions (**d**). Scale bars = 1 cm (**a**-**e** left and middle panels: phosphor screen autoradiography) and 50 μm (**a**-**e** right panels; high resolution nuclear emulsion autoradiography)

Phosphor screen autoradiography experiments revealed strong binding of [F-18]-MK-6240 in the hippocampal formation/EC and frontal, temporal, parietal and occipital cortices from brain slices containing NFT in AD cases (Fig. 1a). This binding was blocked after incubating the slides with 500 nM unlabeled MK-6240, demonstrating the selectivity of the signal. No binding was detected in non-tangle containing cortical regions or in the white matter in AD and control cases (Fig. 1b). MK-6240 binding was also absent in the cerebellum - typically used in neuroimaging studies as a reference region and lacking tangles in AD – and in the basal ganglia (Fig. 1a-f) of all the cases studied in this series. Of note, no detectable MK-6240 binding could be observed in brain slices containing non-PHF tau aggregates from PiD, PSP, CBD and CTE cases (Fig. 1c, e-f) or in a *MAPT*TP301L mutation carrier (Fig. 1d). This favors the idea that MK-6240 binds with significantly stronger affinity and selectivity to tau aggregates containing all six isoforms of tau (3R and 4R) with paired helical filament (PHF) ultrastructure than to tau lesions primarily made of either 3R or 4R isoforms with straight filament ultrastructure. Brain slices from a D23N Iowa APP mutation carrier [29] displaying very severe CAA but no tau aggregates completely lacked [F-18]-MK-6240 autoradiographic signal (Fig. 2a) and were indistinguishable from control brain slices. Brain slices containing TDP-43 inclusions in FTLD-TDP-43 cases (Fig. 2b) and α-synuclein lesions in DLB (Fig. 2c) and MSA (Fig. 2d) cases also lacked detectable [F-18]-MK-6240 binding. A strong off-target binding of [F-18]-MK-6240 was present in midbrain slices containing substantia nigra in all samples examined, regardless of the presence or absence of tau aggregates; this signal was blocked almost completely when incubating the slides with 500 nM unlabeled MK-6240 (Fig. 3a). In addition, [F-18]-MK-6240 off-target binding was also noticed in retinal pigment epithelium, brain metastatic melanoma, brain slices containing parenchymal hemorrhages and extracutaneous meningeal melanocytes (Fig. 3b-f).

**Fig. 4:**
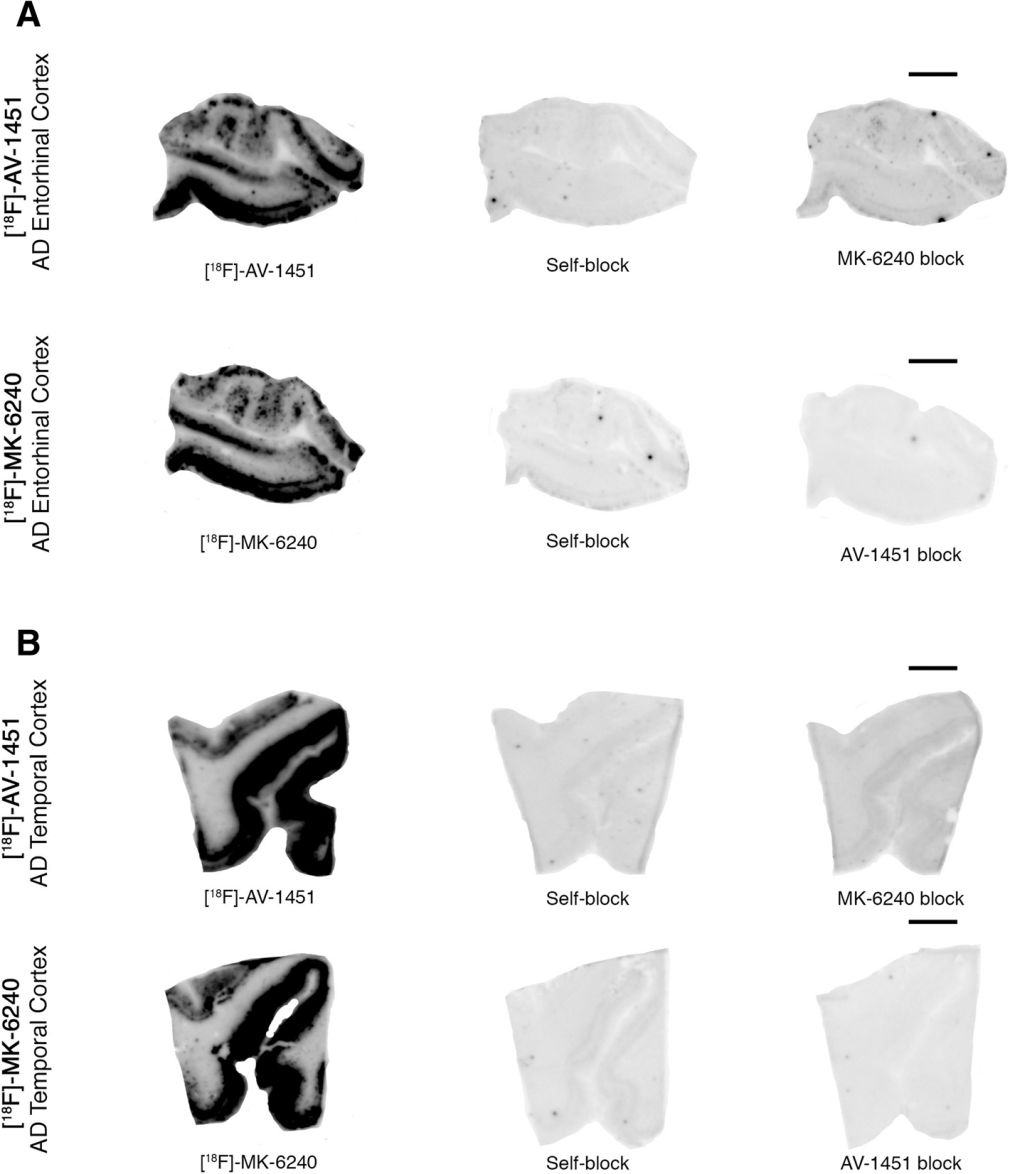
Head-to-head comparison of [F-18]-MK-6240 and [F-18]-AV-1451 phosphor screen autoradiographic binding patterns in adjacent section obtained from the same tissue material containing entorhinal cortex (#13) (**a**) and superior temporal sulcus (#13) (**b**) from AD cases. Both tracers exhibited comparable strong binding in regions containing tangles; MK-6240 signal was blocked by adding 500 nM unlabeled AV-1451 and AV-1451 signal was almost completely blocked by adding 500 nM unlabeled MK-6240. Scale bar = 1 cm

Overall, the [F-18]-MK-6240 autoradiographic specific and off-target binding patterns described above are comparable to those exhibited by [F-18]-AV-1451 that we and others have previously described in detail elsewhere [22]. Of note, [F-18]-MK-6240 binding was blocked after incubating the slides with 500 nM unlabeled AV-1451 and vice versa (Fig. 4).

**Fig. 5:**
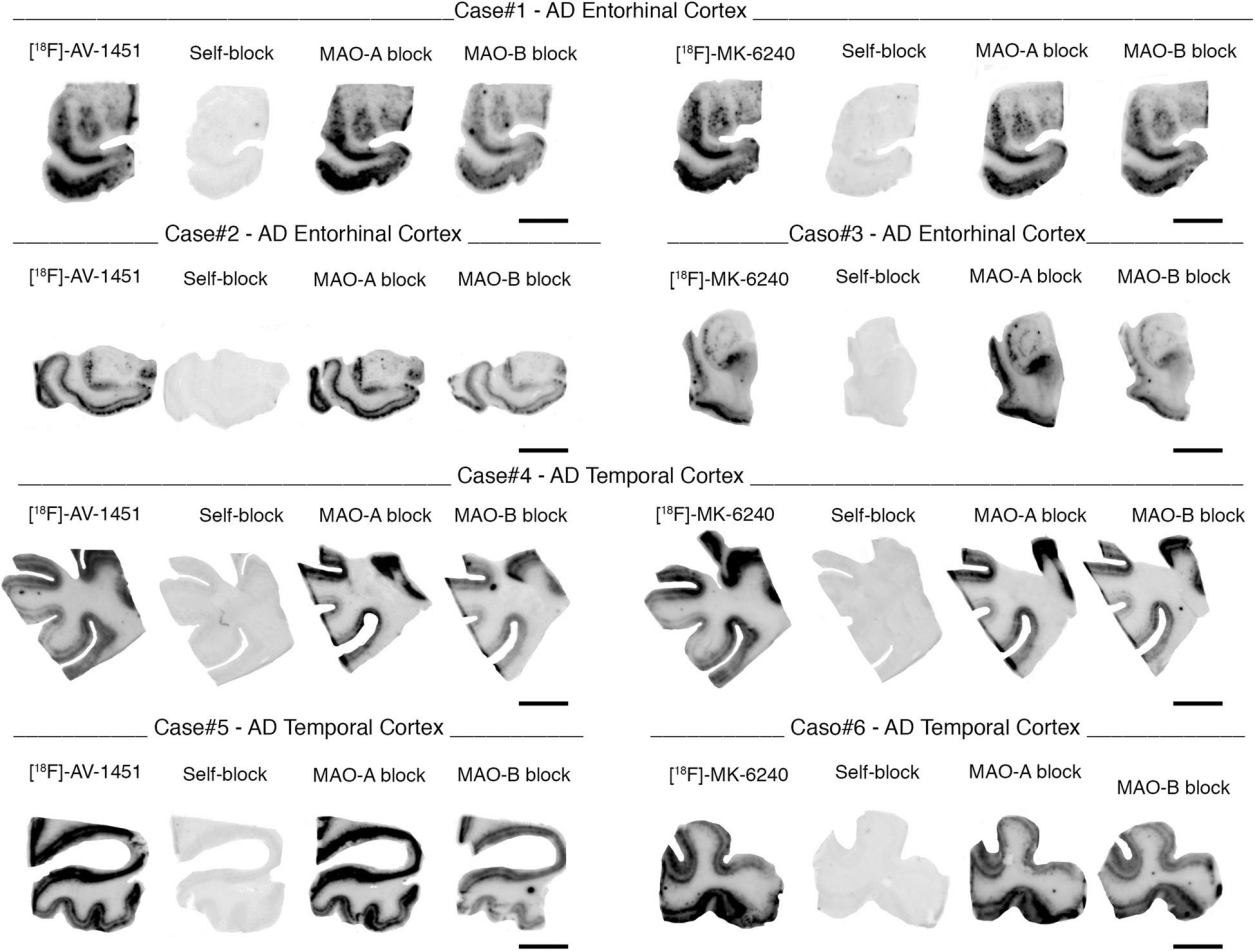
Phosphor screen autoradiography experiments in slices containing entorhinal and temporal cortices from AD cases using competing concentrations of 1 μM clorgyline (a selective MAO-A inhibitor) and deprenyl (MAO-B inhibitor). [F-18]-MK-6240 and [F-18]-AV-1451 binding signals are only weakly displaced (by about 20%) with 1 μM deprenyl (a selective MAO-B inhibitor). When a competing concentration of 1 μM clorgyline (a selective MAO-A inhibitor) was added to the blocking solution, neither [F-18]-MK-6240 nor [F-18]-AV-1451 autoradiographic signal displacement could be detected. Scale bar = 1 cm

Importantly, when a competing concentration of 1 μM clorgyline (a selective MAO-A inhibitor) was added to the blocking solution, neither [F-18]-MK-6240 nor [F-18]-AV-1451 autoradiographic signal displacement could be detected (Fig. 5). [F-18]-MK-6240 and [F-18]-AV-1451 binding signals were only very weakly displaced (by about 20%) with 1 μM deprenyl (a selective MAO-B inhibitor) (Fig. 5), pointing to MAO-B as a low binding affinity site of these two tracers.

### [F-18]-MK-6240 high resolution nuclear emulsion autoradiography and immunohistochemistry

With the purpose of obtaining enough resolution at the cellular level, we dipped adjacent brain slices to those used in phosphor screen autoradiography in a photographic nuclear emulsion. Once the slides are developed, the visualization of silver grains struck by positrons emitted during [F-18] nuclear decay enables precise identification of [F-18]-MK-6240 labeled lesions by optical microscopy.

**Fig. 6:**
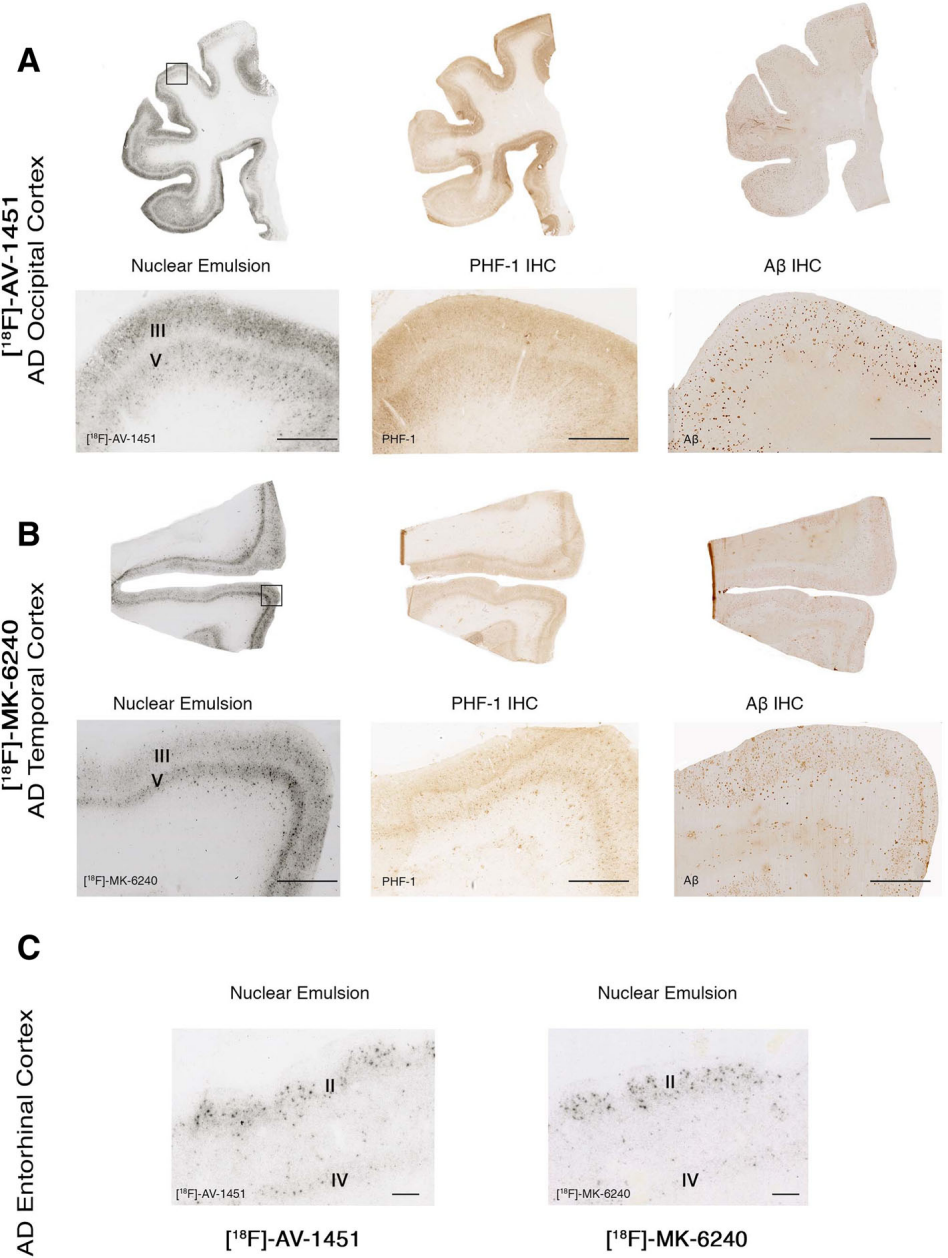
[F-18]-AV-1451 and [F-18]-MK-6240 phosphor screen and high resolution autoradiography photomicrographs of brain slices containing occipital (#13) (**a**), temporal (#9) (**b**) and entorhinal (#14) (**c**) cortices from AD cases (left panels). Middle and right panels (**a** and **b**) show immunostaining of adjacent sections with PHF-1 antibody (kind gift of Dr. Peter Davies) and anti-Aβ antibody (1:500, mouse, clone 6F/3D, Dako), respectively. [F-18]-AV-1451 (left panels **a** and **c**) and [F-18]-MK-6240 (left panel **b** and right panel **c**) high resolution nuclear emulsion autoradiography showed a strong cortical accumulation of silver grains in temporal cortical layers III and V (**a** and **b**), and in layers II and IV of the entorhinal cortex (**c**) in AD brains mirroring the laminar pattern of tangles on adjacent slices as revealed by PHF-1 immunostaining (middle panels) rather than the more scattered plaque distribution pattern revealed by Aβ immunostaining (right panels). Abbreviations: AD = Alzheimer’s disease; IHC = immunohistochemistry. Scale bars = 200 μm (**a** and **b**) 50 μm (**c**)

**Fig. 7:**
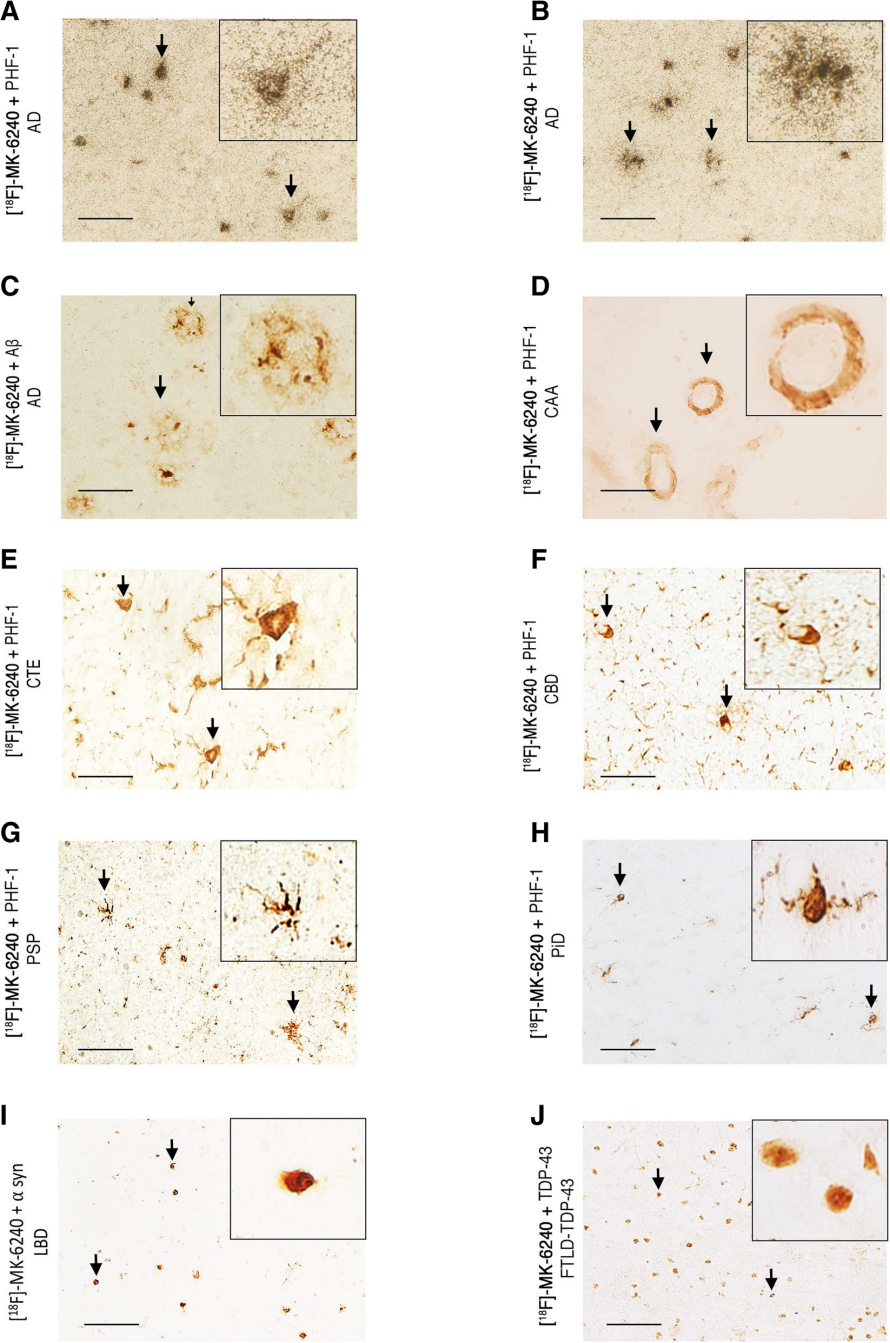
Photomicrographs showing combined [F-18]-MK-6240 high-resolution nuclear emulsion autoradiography followed by immunostaining with appropriate antibodies of brain slices containing frontal and temporal cortices from AD (#10, #16) (**a**-**c**), CAA (#17) (**d**) CTE (#33) (**e**), CBD (#23) (**f**), PSP (#26) (**g**), PiD (#20) (**h**), LBD (#34) (**i**) and FTLD TDP-43 (#39) (**j**). Accumulation of silver grains from the nuclear emulsion colocalized with PHF-1 stained tangles and PHF-tau containing dystrophic neurites around plaques in AD. No detectable accumulations of silver grains were observed in association with Aβ plaques themselves or amyloid-containing vessels in CAA, tau aggregates in CTE, coiled bodies and globose tangles in CBD, astrocytic plaques in PSP, Pick bodies in PiD, Lewy bodies in LBD or TDP-43 inclusions in FTLD TDP-43. Abbreviations: AD = Alzheimer’s disease; CAA = cerebral amyloid angiopathy; CTE = chronic traumatic encephalopathy; CBD = corticobasal degeneration PSP = progressive supranuclear palsy; PiD = Pick’s disease; LBD = Lewy body disease; FTLD TDP-43 = frontotemporal lobar degeneration with TDP-43 inclusions. Scale bar = 50 μm

Using this method, we confirmed the presence of a strong and selective concentration of silver grains in tissue sections from AD cases, reflecting underlying [F-18]-MK-6240 binding in the NFT-containing grey matter with negligible presence of silver grains in the white matter and following a very similar pattern to that observed with [F-18]-AV-1451 (Fig. 6a-b). The silver grain distribution in the hippocampal formation/EC, and frontal, parietal, temporal and occipital cortices in AD brains closely matched the laminar distribution of tangles on adjacent slices as revealed by PHF-1 immunostaining rather than the more scattered plaque distribution pattern revealed by Aβ immunostaining (Fig. 6a-b). Silver grains were particularly abundant in layers III and V of association cortex (Fig. 6a-b) and layers II and IV in the entorhinal cortex (Fig. 6c), matching the well known laminar pattern of NFT in AD [1, 18]. [F-18]-MK-6240 high resolution autoradiography followed by immunostaining with PHF-1 or Aβ antibodies on the same brain slices further confirmed that the lesions labeled by the nuclear emulsion were PHF-tau aggregates, including classic NFT and PHF-tau containing dystrophic neurites around plaques (Fig. 7a-b), but not Aβ plaques themselves (Fig. 7c) or vessels with amyloid deposits (Fig. 7d). NFT-containing slices from AD brains dipped in the nuclear photographic emulsion omitting the incubation with [F-18]-MK-6240 showed no silver grain accumulation and served as negative control (not shown).

Brain slices from control brains free of tau aggregates did not show accumulation of silver grains in any of regions examined (data not shown). Negligible numbers of silver grains were observed colocalizing with tau aggregates in CTE, CBD, PSP and PiD cases (Fig. 7e-h). No silver grains were observed either colocalizing with α-synuclein or TDP-43 containing inclusions (Fig. 7i-j).

Of note, neuromelanin-containing neurons in the substantia nigra pars compacta (Fig. 3a), retinal pigment epithelium (RPE) cells (Fig. 3b), tumor cells of metastatic melanoma (Fig. 3c) and leptomeningeal melanocytes (Fig. 3e), consistently demonstrated a robust concentration of silver grains confirming off-target binding of MK-6240 to neuromelanin- and melanin-containing cells. Weaker concentrations of silver grains were also observed colocalizing with parenchymal hemorrhages (Fig. 3d), pointing to some additional [F-18]-MK-6240 off-target binding to blood components.

## Discussion

Recently multiple novel PET tracers have been reported, tailored to allow detection of tau pathology in the human living brain. The status quo is largely to use PET ligands in patients carrying a particular clinical diagnosis and then simply accept, in a somewhat circular fashion, that what the scan shows is representative of the underlying disease process. However, the correct identification of biological targets of imaging agents is an essential requirement for considering them as disease-specific and progression-specific biomarkers. Validating the underlying neuropathological binding substrate/s and identifying potential off-target binding of these novel tau ligands is critical for the accurate interpretation of their in vivo PET imaging behavior. We have carefully characterized the autoradiographic binding patterns of novel tau tracer [F-18]-MK-6240 in a collection of postmortem tissue samples representing a broad spectrum of neurodegenerative disorders. Our observations derived from combined [F-18]-MK-6240 sensitive autoradiography and immunohistochemistry, using this compound at similar concentrations used in vivo for PET studies, indicate that MK-6240 has high binding affinity for tau aggregates in AD brain tissue, but does not seem to bind to a significant extent to neuronal and glial tau aggregates in non-AD tauopathies such as PiD, PSP, CBD or CTE, or to Aβ, α-synuclein or TDP-43-containing lesions. These findings strongly suggest that tau in tangles of AD has a unique conformation that is recognized by this tracer (in keeping with the selection process for development of this compound as a lead imaging agent). Strong binding of [F-18]-MK-6240 to neuromelanin- and melanin-containing cells and some weaker binding to brain hemorrhagic lesions was also identified, pointing to these substrates as off-target binding sites of MK-6240. Overall, [F-18]-MK-6240 autoradiographic binding patterns closely resembled those of tau PET tracer [F-18]-AV-1451. Importantly, [F-18]-MK-6240 and [F-18]-AV-1451 binding signals were only very weakly displaced using autoradiography competition with unlabeled selective MAO-B inhibitor deprenyl, suggesting that these two tracers have low binding affinity for MAO enzymes in the human brain.

MK-6240 was identified as a potential imaging agent by screening using cortical homogenates from AD tissue rich in NFT as the binding target and an amyloid plaque tracer for counter-screen [15]. Based on this, we anticipated that MK-6240 would preferentially bind to tau lesions in the form of NFT in AD over other tau aggregates in non-AD tauopathies or lesions primarily made of Aβ, α-synuclein or TDP-43. To date, only very limited data from human in vivo [F-18]-MK-6240 PET imaging studies have been published [2, 19]. Results from these early studies point to a promising 2- to 3-fold higher in vivo [F-18]-MK-6240 retention in neocortical and medial temporal brain regions of AD patients compared to elderly cognitively normal individuals [19]. Because this ligand is already being incorporated into clinical trial research, validation studies such as this paper are absolutely critical to evaluate the potential usefulness of this ligand as a reliable marker of human brain tau lesions. In an attempt to advance towards that goal, we applied [F-18]-MK-6240 phosphor screen and high resolution autoradiography to the study of a series of autopsy samples from individuals with a definitive diagnosis of AD, PiD, PSP, CBD, CTE, CAA, FTLD-TDP-43, DLB, and control brains free of neurodegenerative pathology. Our results confirmed that while [F-18]-MK-6240 avidly bound to PHF-tangle containing slices from AD brains, it did not bind to a significant extent to tau-containing lesions in slices from non-AD tauopathy brains, suggesting that this tracer has higher affinity and selectivity for PHF-tau over tau aggregates with a primarily straight filament ultrastructure, and thus raising reasonable doubts about the potential value of this ligand as a biomarker of tau pathology in non-AD tauopathies. The regional and laminar autoradiographic patterns of distribution of [F-18]-MK-6240, as revealed by the combination of autoradiography using a fine grain nuclear emulsion and immunohistochemistry, closely matched those of classic PHF-tangles in AD brains [1, 18]. Using this method, we confirmed that [F-18]-MK-6240-labeled lesions were NFT, suggesting that these lesions are the main pathological substrate of [F-18]-MK-6240 binding. The microscopic examination of diffuse plaques, CAA, α-synuclein and TDP-43 aggregates confirmed the absence of detectable [F-18]-MK-6240 binding to these lesions, favoring the relative selectivity of [F-18]-MK-6240 for NFT over β-amyloid plaques and other abnormal protein aggregates with a β-pleated sheet conformation.

Our data also establish that MK-6240 is not fully selective for PHF-tau deposits. Similarly to AV-1451, MK-6240 exhibits strong off-target binding to neuromelanin- and melanin-containing cells including pigmented neurons in the substantia nigra (regardless of the presence or absence of nigral tau pathology), leptomeningeal melanocytes, metastatic melanoma and retinal pigment epithelium, with some weaker off-target binding to brain hemorrhages as well. This is something relevant for the correct interpretation of [F-18]-MK-6240 in vivo imaging depending for example on the relative abundance and distribution of leptomeningeal melanocytes across different individuals [10], the possibility of focal artifactual increases in the density of these cells due to regional cortical atrophy, or the presence of concomitant brain hemorrhagic lesions.

One of the first generation tau PET tracers, THK-5351, has been recently found to demonstrate high binding affinity to MAO-B [13, 24], seriously compromising its value as a tau-specific tracer and increasing the need for alternative tau-specific imaging agents. To date, studies on potential non-specific binding of AV-1451 to MAO enzymes are scarce and have yielded conflicting results. A recent study by Vermeiren and colleagues suggested that H3-AV-1451 binds with similar nanomolar affinity to tau fibrils and MAO-A and B enzymes in brain homogenates isolated from AD or PSP patients as well as those devoid of tau pathology [30]. Merck’s researchers also reported high affinity displacement of 3H-AV-1451 binding, but not of 3H-MK-6240, in some non-AD brain homogenates in the presence of selective MAO-A inhibitor clorgyline. By contrary, Hansen and colleagues found that MAO-B inhibitors did not block in vivo [F-18]-AV-1451 binding in a series of 16 of 27 PD patients receiving MAO-B inhibitors at the time of scan [12]. In agreement with these results, Lemoine et al. reported that AV-1451 shows ten times lower affinity to MAO-B when compared to THK-5351 in in vitro assays [17]. Consistent with these observations, our data derived from [F-18]-MK-6240 and [F-18]-AV-1451 autoradiography experiments in the presence of selective MAO-A and MAO-B inhibitors point to a low binding affinity of both tracers for MAO enzymes. Studies using the specific enzymatic inhibitors do not exclude interaction of MK-6240 with MAO isoforms at regions removed from the active site. The discrepancies among the different studies could stem from the different techniques and isotope labeling used in each case e.g. in vitro binding assays in brain homogenates vs. autoradiography assays in tissue slices, and labeling of the tau tracer with H-3 vs. F-18. Our previous experience with H-3-AV-1451 in vitro binding assays in brain homogenates suggested that the signal-to background noise ratio when using this method was relatively low compared to [F-18]-AV-1451 autoradiographic techniques; something that could be due, at least in part, to off-target binding to sample components such as blood.

## Conclusions

In conclusion, all together our results show that MK-6240 exhibits a nearly identical binding profile to AV-1451 holding promise as a potential surrogate marker for the in vivo detection of neurofibrillary tangles in AD, while still having various forms of off-target binding that need to be considered when interpreting in vivo imaging findings. The utility of MK-6240 for the reliable in vivo detection of tau aggregates in non-AD tauopathies, however, seems very limited. Both, MK-6240 and AV-1451, as opposed to other tracers like THK-5351, seem to exhibit relatively low binding affinity for MAO enzymes. Future imaging-pathological correlation studies on postmortem material from patients scanned while alive will provide additional information on the utility of these two tracers for the reliable quantification of NFT burden in AD and disease progression tracking by in vivo neuroimaging, as well as their potential usefulness when testing therapeutic approaches aimed at decreasing or halting the progression of tau aggregation in AD.
